# Characterization of carbapenem-resistant *Klebsiella pneumoniae* in bloodstream infections: antibiotic resistance, virulence, and treatment strategies

**DOI:** 10.3389/fcimb.2025.1541704

**Published:** 2025-03-07

**Authors:** Chenglin Zhong, Shaohua Lin, Zeqi Li, Xuejing Yang

**Affiliations:** The First Affiliated Hospital of Zhejiang Chinese Medical University (Zhejiang Provincial Hospital of Traditional Chinese Medicine), Hangzhou, Zhejiang, China

**Keywords:** CRKP, bloodstream infections, clinical features, multidrug resistance, risk factors, berberine hydrochloride

## Abstract

**Background:**

Carbapenem-resistant *Klebsiella pneumoniae* (CRKP) infections pose a major clinical challenge due to multidrug resistance. This study evaluated the clinical features, antibiotic resistance mechanisms, virulence factors, and the potential therapeutic impact of berberine hydrochloride (a traditional Chinese medicine) in CRKP infections.

**Methods:**

Ninety-four CRKP isolates from bloodstream infections at the First Affiliated Hospital of Zhejiang Chinese Medical University were characterized for carbapenemase genes, antibiotic susceptibility, and virulence determinants. Clinical data were analyzed to identify risk factors for CRKP infection, and the *in vitro* antibacterial activity of berberine hydrochloride was assessed.

**Results:**

Most of the isolates (71.3%) were from the intensive care unit (ICU) patients. The *bla*_KPC_ gene was the predominant resistance mechanism (62.77%), while the virulence genes *uge* (93.62%) and *wabG* (92.55%) were highly prevalent. ICU admission, male sex, respiratory diseases, invasive procedures, prior use of third-generation cephalosporinase inhibitors, and absence of traditional Chinese medicine treatment were linked to poorer outcomes. Importantly, berberine hydrochloride inhibited CRKP growth *in vitro*, with a minimum inhibitory concentration (MIC) of 125 mg/mL.

**Conclusion:**

Our study reveals the multifaceted resistance and virulence profiles of CRKP in bloodstream infections and highlights the potential clinical value of berberine hydrochloride as an adjunctive therapeutic agent. These findings support further clinical investigations into incorporating traditional Chinese medicine to improve outcomes in patients with CRKP bloodstream infections.

## Introduction

*Klebsiella pneumoniae* (KP) is a significant gram-negative opportunistic pathogen that colonizes human skin, the respiratory tract, and the intestine. A weakened defense response or compromised natural barriers can lead to various infections, such as those of the urinary tract and bloodstream, pneumonia, and liver abscesses (Jondle et al., 2018; Aghamohammad et al., 2020). Data from the China Antimicrobial Surveillance System from 2014-2019 underscore the prevalence of KP, which constitutes 10.2% of the bacteria isolated from blood samples (China Antimicrobial Resistance Surveillance System, 2021). Moreover, the resistance rates to imipenem and meropenem range from 6.6% to 11.7%, showing an increasing trend over the years (China Antimicrobial Resistance Surveillance System, 2021), thereby posing a significant challenge to their clinical treatment.

In recent years, the widespread use of broad-spectrum antibiotics has led to the emergence of highly drug-resistant and pathogenic strains of KP. The emergence of carbapenem-resistant Enterobacteriaceae (CRE) represents a critical challenge in clinical anti-infection therapy (Barnes et al., 2019). CRE encompasses a spectrum of resistance mechanisms, including β-lactamase production, the modification of porins, and changes in bacterial cell membrane permeability (Anderson and Boerlin, 2020). These mechanisms confer resistance against conventional antibiotics, thus complicating treatment strategies. Enterobacter species can produce carbapenemase and extended-spectrum β-lactamase (ESBL) enzymes, which confer resistance to third-generation antibiotics, thereby further increasing the therapeutic challenge. Ambler’s molecular classification divides carbapenemases into three groups: class A (carbapenemases, such as KPC and GES), class B (metallo β-carbapenemases or MBLs, such as VIM, IMP, and NDM), and class D (oxacillinase or OXA, such as OXA-48) (Bush et al., 1995; Queenan and Bush, 2007; Bush and Jacoby, 2010). Additionally, the emergence of hypervirulent KP (hvKP) strains has increased mortality rates through more challenging treatments (Shon et al., 2013; Dai and Hu, 2022; Han et al., 2022; Yang et al., 2022). Among these strains, virulence genes such as *uge* and *wabG* are commonly found, contributing to their high pathogenicity (Remya et al., 2019; Wang et al., 2020; Kot et al., 2023). The high pathogenicity of these strains is attributed to virulence genes such as *uge* and *wabG*, which along with other factors (such as capsular polysaccharides and biofilm formation), play crucial roles in the virulence of KP (Srinivasan et al., 2012; Dunstan et al., 2023).

Rising infections and the emergence of CRKP strains have intensified the clinical challenge of managing multidrug-resistant pathogens. Previous studies integrating traditional Chinese and Western medicine have demonstrated that compounds such as scutellarin can block biofilm formation (Yin et al., 2016) and that glucoside B can inhibit bacterial efflux pumping activity (Zhong Haiqin and Ting, 2013), thereby increasing antimicrobial efficacy. Despite these promising findings, the therapeutic potential of berberine hydrochloride (a well-established traditional Chinese medicine) remains underexplored in the context of CRKP infections. Given the limited effectiveness of conventional antibiotics against CRKP, we hypothesize that berberine hydrochloride can mitigate key resistance mechanisms and improve antibacterial outcomes. To test this hypothesis, our study aimed to evaluate the *in vitro* efficacy of berberine hydrochloride against CRKP isolates and elucidate its role as an adjunct treatment strategy, ultimately bridging the gap between carbapenem resistance challenges and innovative, integrative therapeutic approaches.

In this study, we comprehensively evaluated the clinical features, antibiotic resistance profiles, virulence-associated molecular characteristics, and risk factors for CRKP bloodstream infections. In parallel, we assessed the *in vitro* efficacy of berberine hydrochloride as an adjunctive therapeutic strategy. These findings not only provide a robust theoretical foundation for the prevention, control, and clinical management of CRKP infections but also underscore the critical role of traditional Chinese medicine in enhancing patient outcomes.

## Materials and methods

### Data collection

A total of 94 nonrepetitive clinical CRKP strains were isolated from the blood samples of 94 patients (60 males and 34 females) at the First Affiliated Hospital of Zhejiang Chinese Medical University from June 2017 to October 2022. The inclusion criteria were as follows: (1) the isolate was confirmed as *Klebsiella pneumoniae* with resistance to carbapenems according to established CLSI guidelines, (2) the isolate was obtained from a patient diagnosed with a clinically significant bloodstream infection, and (3) complete clinical and microbiological data were available for analysis. The exclusion criteria included (1) duplicate strains from the same patient, defined as multiple isolates with identical phenotypic and genotypic profiles during the same hospitalization period; (2) isolates that did not meet the laboratory criteria for CRKP on the basis of their resistance profiles; and (3) isolates with incomplete or missing clinical information. Patient data, including sex, age, duration of hospitalization, underlying diseases, antibiotic treatment, traditional Chinese medicine treatment, history of invasive procedures, and patient prognosis, were collected to investigate the risk factors associated with CRKP bloodstream infections.

### Antibiotic susceptibility testing

All CRKP isolates were identified via matrix-assisted laser desorption ionization time-of-flight mass spectrometry (MALDI-TOF MS, Bruker Daltonics, Bremen, Germany). Antibiotic susceptibility testing of the 94 clinical CRKP isolates was conducted via the Vitek-2 Compact system (Biomerieux, Marcy l’Etoile, Lyon, France). The tested antibiotics included amoxicillin, ampicillin, ceftazidime, cefepime, cefoperazone, ceftriaxone, ertapenem, imipenem, meropenem, piperacillin, ciprofloxacin, levofloxacin, amikacin, gentamycin, tobramycin, polymyxin, tigecycline, nitrofurantoin, aztreonam and puromycin. The minimum inhibitory concentrations (MICs) of meropenem, imipenem, and ertapenem were determined via the broth microdilution method according to the Clinical and Laboratory Standards Institute (CLSI) 2020-M100 guidelines. Quality control strains, including *Klebsiella pneumoniae* (ATCC700603), *Pseudomonas aeruginosa* (ATCC27853), *Staphylococcus aureus* (ATCC25913), and *Escherichia coli* (ATCC25922), were obtained from the China National Health Inspection Center.

### Carbapenem resistance detection and virulence phenotyping of CRKP isolates

The carbapenem-resistant phenotype was confirmed via carbapenem inhibitor enhancement experiments conducted via the disc diffusion method (Hua. et al., 2020). Briefly, a single colony of the CRKP isolate was diluted to a bacterial suspension (0.5 McFarland standard) using sterile saline, followed by spreading onto Mueller Hinton (MH) agar plates. Four discs of carbapenem (either imipenem or meropenem) were placed on the agar surface. One disc served as a control, while the second, third, and fourth discs were treated with 10 μL of 50 mg/ml APB (3-aminophenyl boronic acid hydrochloride), 10 μL of 0.5 M EDTA (ethylenediaminetetraacetic acid disodium salt dehydrate), and 10 μL of 50 mg/ml APB mixed with 10 μL of 0.5 M EDTA solution, respectively. The plates were then incubated overnight at 35°C, after which the diameter of the antibacterial zone around the paper discs was measured. The results were interpreted according to established guidelines.

Next, the mucous phenotype was confirmed via a wire drawing experiment. A single colony from the agar plate was picked via a sterile inoculation ring. The mucus filament length exceeding 5 mm in three independent replicates represented a high mucous phenotype. Furthermore, serum collected from healthy individuals was used for the serum resistance test. The bacterial suspension from the overnight culture was diluted with Luria-Bertani (LB) broth (0.5 McFarland standard), mixed with serum, and incubated at 35°C for 0, 1, 2, and 3 h. After tenfold dilution, the mixture was spread onto MH agar plates and incubated overnight. Serum resistance was evaluated on the basis of colony-forming unit (CFU) counts (Chen and Kreiswirth, 2018; Mitra et al., 2019).

Biofilm formation experiments were conducted via the crystal violet staining method (Naparstek et al., 2014). The diluted bacterial suspension was added to a 96-well plate, which was subsequently sealed and incubated overnight at 35°C. After washing three times with neutral phosphate-buffered saline (PBS), 1% crystal violet was added for 15 min. After repeatedly rinsing with water and drying, anhydrous ethanol was added to dissolve the crystal violet, and the optical density (OD) was measured at 590 nm. The OD values indicate the extent of adhesion of the strain biofilm to the contact surface of the 96-well polystyrene plate.

### Detection of carbapenem resistance genes and virulence-associated genes

Genomic DNA isolation from CRKP isolates was performed via the thermal lysis method, followed by the detection of genes conferring carbapenem resistance and virulence via polymerase chain reaction (PCR). The primers used were designed according to previous studies (Candan and Aksöz, 2015; Makharita et al., 2020). The detailed primer sequences and the PCR parameters used for this study are listed in **Supplementary Tables S1**-**S4**. The positive PCR products were subsequently sequenced (Shanghai Sangon Biotech Co., Ltd.), and the results were analyzed via the BLAST tool available at NCBI.

### *In vitro* minimum inhibitory concentration (MIC) assay

Berberine hydrochloride was serially diluted in MH broth to final concentrations of 7.81, 15.63, 31.25, 62.5, 125, 250, and 500 mg/mL. These concentration ranges were selected on the basis of preliminary experiments and literature reports indicating effective inhibitory levels against multidrug-resistant bacteria. CRKP strains were cultured in MH broth and exposed to the various concentrations of berberine hydrochloride, whereas the control groups received an equal volume of MH broth. The cultures were incubated at 37°C for 24 h, with bacterial growth was monitored by measuring the OD at 490 nm every 2 h. After the 24-hours incubation period, aliquots from each culture were spread onto MH agar plates and further incubated for 24 h to assess colony formation. The minimum inhibitory concentration (MIC) was defined as the lowest concentration of berberine hydrochloride that completely inhibited bacterial growth, as evidenced by the absence of colonies on the agar plates.

### Statistical analysis

All the figures and graphs were prepared via GraphPad Prism 9.5 software. The antibiotic susceptibility profiles of the CRKP strains were analyzed via WHONET 5.6, and all the statistical analyses were performed with SPSS 25.0. For univariate analysis, the χ^2^ test and *t*-test were employed for qualitative and quantitative data, respectively, with a *P-*value of < 0.05 considered statistically significant. Variables meeting this threshold were then included in a forward binary logistic regression model to calculate the odds ratios (ORs) of related risk factors. Additionally, comparisons among multiple groups were carried out via one-way analysis of variance (ANOVA) followed by Tukey’s *post hoc* test.

## Results

### Department distribution of clinical CRKP isolate data

**Figure 1:**
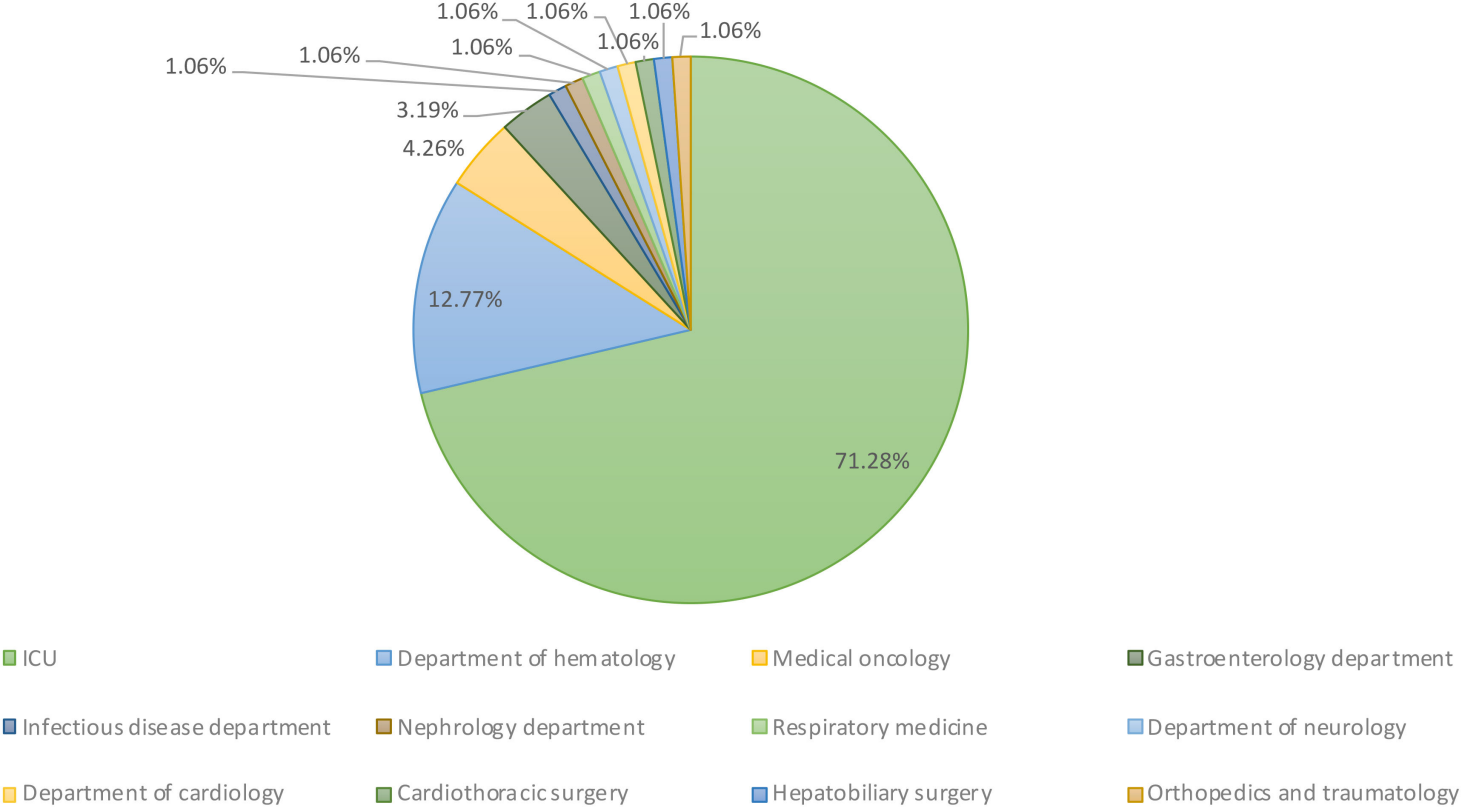
Department distribution of 94 clinical CRKP bloodstream infection strains. These strains were collected from a total of 12 different departments within the hospital setting.

All the nonrepetitive clinical CRKP strains (94 in total) were isolated from blood samples collected from patients admitted to 12 different departments, including the ICU (71.28%, n = 67), hematology (12.77%, n = 12), medical oncology (4.26%, n = 4), gastroenterology (3.19%, n = 3), and other departments (8.48%, n = 8) (**Figure 1**).

### Antibiotic susceptibility profiles

All the CRKP isolates were identified as multidrug-resistant strains, posing a major challenge for both treatment and infection control in healthcare settings. The antibiotic susceptibility of these strains was evaluated against 20 commonly used clinical antibiotics. The results revealed that the isolates exhibited 100% resistance to piperacillin, meropenem, ertapenem, ampicillin, ceftriaxone, and amoxicillin (**Table 1**). The resistance rates ranged from 75% to 99% for cefoperazone, ceftazidime, cefepime, aztreonam, tobramycin, ciprofloxacin, levofloxacin, imipenem, nitrofurantoin, and gentamycin (**Table 1**). In contrast, lower resistance rates (<75%) were observed for puromycin, polymyxin, tigecycline, and amikacin (**Table 1**). Notably, CRKP strains presented high sensitivity to polymyxin, with a resistance rate of only 5.32% (**Table 1**), underscoring its potential as a last-resort therapeutic agent. Furthermore, the high resistance levels, particularly in high-risk hospital areas such as the ICU, suggest that these isolates may be spreading clonally within the hospital environment.

**Table 1:** Antibiotic susceptibility profiles of 94 clinical CRKP strains.

Antibiotics	No.	%R	No.	%I	No.	%S
β-lactams						
Amoxicillin	94	100	0	0	0	0
Ampicillin	94	100	0	0	0	0
Ceftazidime	91	96.81	1	1.06	2	2.13
Cefepime	82	87.23	2	2.30	10	10.64
Cefoperazone	93	98.94	0	0	1	1.06
Ceftriaxone	94	100	0	0	0	0
Ertapenem	94	100	0	0	0	0
Imipenem	92	97.87	0	0	2	2.13
Meropenem	94	100	0	0	0	0
Piperacillin	94	100	0	0	0	0
Aztreonam	90	95.74	1	1.06	3	3.19
Quinolones						
Ciprofloxacin	84	89.36	10	10.64	0	0
Levofloxacin	83	88.30	6	6.38	5	5.32
Aminoglycosides						
Amikacin	69	73.40	24	25.53	1	1.06
Gentamycin	79	84.04	15	15.96	0	0
Tobramycin	73	77.66	16	17.02	5	5.32
Polypeptide						
Polymyxin	5	5.32	89	94.68	0	0
Tetracyclines						
Tigecycline	49	52.13	39	41.49	6	6.38
Nitrofurans						
Nitrofurantoin	80	85.11	1	1.06	13	13.83
Others						
Puromycin	63	67.02	30	31.91	1	1.06

R, resistant; I, intermediate; S, susceptible.

### Carbapenem-resistant genotypes and phenotype analysis of CRKP strains

Our sequencing results confirmed the genotypic distribution of carbapenem resistance among the CRKP isolates. The *bla*_KPC_ was the most prevalent and was detected in 59 strains (62.77%), followed by the *bla*_NDM_ gene in 14 strains (14.89%) (**Table 2**). Additionally, minor carbapenemase genes, including *bla*_VEB_, *bla*_OXA-48_, *bla*_GES_, and *bla*_IMP_, were identified, while the *bla*_VIM_ gene was notably absent (**Table 2**), suggesting the presence of alternative resistance mechanisms.

**Table 2:** Detection of carbapenemase genes and virulence genes in 94 CRKP strains.

Genotypes	No.	%
Carbapenemase genes		
*bla*_KPC_	59	62.77
*bla*_NDM_	14	14.89
*bla*_VEB_	5	5.32
*bla*_OXA-48_	2	2.13
*bla*_GES_	2	2.13
*bla*_IMP_	1	1.06
*bla*_VIM_	0	0
Virulence genes		
*uge*	88	93.62
*wabG*	87	92.55
*fimH*	62	65.96
*rmpA*	42	44.68
*iroN*	30	31.91
*aerobactin*	9	9.57
*alls*	2	2.13
*mrkD*	2	2.13
*wcaG*	1	1.06
*iutA*	0	0
*entB*	0	0
*magA*	0	0

Phenotypic analysis of carbapenemase activity further revealed that 77.6% of the strains produced class A serine proteases, 10.6% produced class B metalloenzymes, and 11.7% coproduced both types of carbapenemases (**Table 2**). These findings underscore the genetic diversity of CRKP strains and their potential for widespread dissemination in healthcare settings. The predominance of *bla*_KPC_, in particular, suggests a high risk of hospital transmission due to its association with clonal expansion in nosocomial outbreaks.

### Virulence-associated genotypes and phenotype analysis of CRKP strains

We next analyzed the distribution of virulence genes among the CRKP isolates to elucidate their potential impact on hospital transmission and clinical outcomes. The results revealed that the virulence genes *uge* and *wabG* were the most prevalent, with positive rates of 93.62% and 92.55%, respectively (**Table 2**). These genes, known to be involved in capsule biosynthesis and immune evasion, may facilitate persistent colonization in hospital environments. Other virulence determinants were detected at lower frequencies: *fimH* (65.96%), *rmpA* (44.68%), *iroN* (31.91%), *aerobactin* (9.57%), *alls* (2.13%), *mrkD* (2.13%), and *wcaG* (1.06%) (**Table 2**). Notably, *iutA*, *entB*, and *magA* were absent from our isolates (**Table 2**).

In addition to genotypic profiling, we also assessed the phenotypic virulence characteristics of the CRKP strains. Only 15 strains (15.79%) presented a hypermucoviscous phenotype, whereas the remaining strains presented a normal phenotype (**Table 3**). The capsular serotype gene was detected in a mere 3 strains (3.19%), with the K1 and K2 serotypes found in two and one strains, respectively; the other major serotypes (K1, K2, K5, K20, K54, and K57) were not identified in the remaining isolates (**Table 3**). Furthermore, serum resistance and biofilm formation assays provided additional insights into the potential for nosocomial dissemination. Among the 94 strains, 50 (53.9%) were serum resistant, 32 (34.04%) were moderately sensitive, and 12 (12.77%) were highly sensitive (**Table 3**). In terms of biofilm formation, 6 strains (6.38%) presented strong biofilm-forming ability, 35 strains (37.23%) presented intermediate ability, and 53 strains (56.38%) presented weak biofilm formation (**Table 3**). Taken together, the high prevalence of virulence genes such as *wabG*, *fimH*, *rmpA*, and *iroN* among CRKP bloodstream isolates highlights their potential role in facilitating persistent colonization and transmission within hospital settings. Although only a minority of isolates exhibited high virulence-associated phenotypes (e.g., hypermucoviscosity and capsular serotypes K1/K2), the presence of these factors may contribute to severe infections and nosocomial outbreaks.

**Table 3:** Phenotype detection of 94 clinical CRKP strains.

Phenotypes	No.	%
Carbapenem-resistant		
class A	73	77.66
class B	10	10.64
class A and B	11	11.7
Mucous phenotype		
high mucous	15	15.79
normal	79	84.04
Serum resistant		
serum resistant	50	53.19
intermediate sensitive	32	34.04
highly sensitive	12	12.77
Capsular serotype		
K1	2	2.13
K2	1	1.06
K5	0	0
K20	0	0
K54	0	0
K57	0	0
Biofilm formation capacity		
strong	6	6.38
intermediate	35	37.23
weak	53	56.38

The carbapenem-resistant phenotypes of CRKP strains can be categorized into three classes: Class A indicates the production of class A serine proteases; Class B indicates the production of class B metalloenzymes; and Class A and B indicate the production of both class A and class B enzymes.

### Risk factors and multivariate analysis of CRKP bloodstream infections

The results revealed statistically significant differences in several factors associated with poor prognosis in patients with CRKP bloodstream infections. These factors included admission to the ICU (*P* < 0.01), male sex (*P* = 0.022), absence of traditional Chinese medicine treatment (*P* = 0.09), presence of respiratory disease (*P* = 0.010), history of invasive procedures (*P* < 0.001), and treatment with third-generation cephalosporin enzyme inhibitors (*P* = 0.005) (**Table 4**). These findings suggest that these variables could serve as risk factors for poor prognosis in patients with CRKP bloodstream infections. For invasive procedures, four specific factors, namely, venous intubation, nasogastric tube placement, hemodialysis, and arterial catheterization, were identified as significant risk factors for poor prognosis in patients with CRKP bloodstream infections (*P* < 0.05) (**Table 4**).

**Table 4:** Clinical features of 94 CRKP bloodstream infection patients.

Variables	Total n= 94 (%)	Poor prognosis		*P*
		Yes, n= 66 (%)	No, n= 28 (%)	
General information				
Gender: male	60 (63.83%)	47 (71.21%)	13 (46.43%)	0.022^*^
Age: ≥ 60	72 (76.60%)	54 (81.82%)	18 (64.28%)	0.066
ICU	65 (69.15%)	53 (80.30%)	12 (42.86%)	0.000^**^
Concomitant disease				
Hypertension	53 (56.38%)	36 (54.54%)	17 (60.71%)	0.457
Respiratory diseases	27 (28.72%)	25 (37.88%)	2 (7.14%)	0.010*
Heart failure	1 (1.06%)	1 (1.52%)	0	1.000^b^
Heart disease	18 (19.15%)	12 (18.18%)	6 (21.43%)	0.714
Hematological-malignancies	14 (14.89%)	10 (15.15%)	4 (14.28%)	1.000^a^
Invasive procedure				
Venous cannula	48 (51.06%)	43 (65.15%)	5 (17.86%)	0.000^**^
Nasogastric tube	36 (38.30%)	30 (45.45%)	6 (21.43%)	0.028^*^
Hemodialysis	16 (17.02%)	16 (24.24%)	0	0.002^** b^
Artery place pipe	15 (15.96%)	15 (22.73%)	0	0.004^** b^
**Traditional Chinese medicine treatment**	29 (30.85%)	15 (22.73%)	14 (50.00%)	0.009^**^
**Third generation cephalosporin enzyme inhibitor treatment**	9 (9.57%)	5 (7.58%)	4 (14.28%)	0.005^**^

^a^Chi-square test continuity correction; ^b^Fisher exact concept method; bold text indicates **P < 0.01; *P < 0.05.

Furthermore, the results of multivariate binary logistic regression analysis demonstrated that treatment with traditional Chinese medicine had a significant positive effect on poor prognosis (OR, 4.025; 95% confidence interval (CI), 1.018-15.98), indicating a beneficial effect on patient outcomes (**Table 5**). Conversely, respiratory diseases (OR, 0.099; 95% CI, 0.014-0.701), venous intubation (OR, 0.058; 95% CI, 0.016-0.214), and treatment with third-generation cephalosporin enzyme inhibitors (OR, 0.042; 95% CI, 0.050-0.945) had a significant negative impact on prognosis (**Table 5**). Overall, traditional Chinese medicine, along with specific patient characteristics and treatments, serves as a crucial independent risk factor affecting the prognosis of CRKP infections. These findings align with those of previous studies and underscore the importance of these factors in the clinical management and treatment of CRKP bloodstream infections.

**Table 5:** Multivariate binary logistic regression analysis for poor prognosis in patients with CRKP infection.

Risk factors	Wald χ^2^	*P*	OR	95% CI
Traditional Chinese medicine treatment	3.941	0.047	4.025	1.018-15.918
Respiratory diseases	5.363	0.021	0.099	0.014-0.701
Venous cannula	18.253	<0.001	0.058	0.016-0.214
Third-generation cephalosporin enzyme inhibitor treatment	4.141	0.042	0.218	0.05-0.945
Intercept	2.493	0.114	2.197	0.827-5.839

The dependent variable is poor prognosis; McFadden R^2^ = 0.420; Cox & Snell R^2^ = 0.400; Nagelkerke R^2^ = 0.568.

### Berberine hydrochloride inhibited CRKP growth *in vitro*

**Figure 2:**
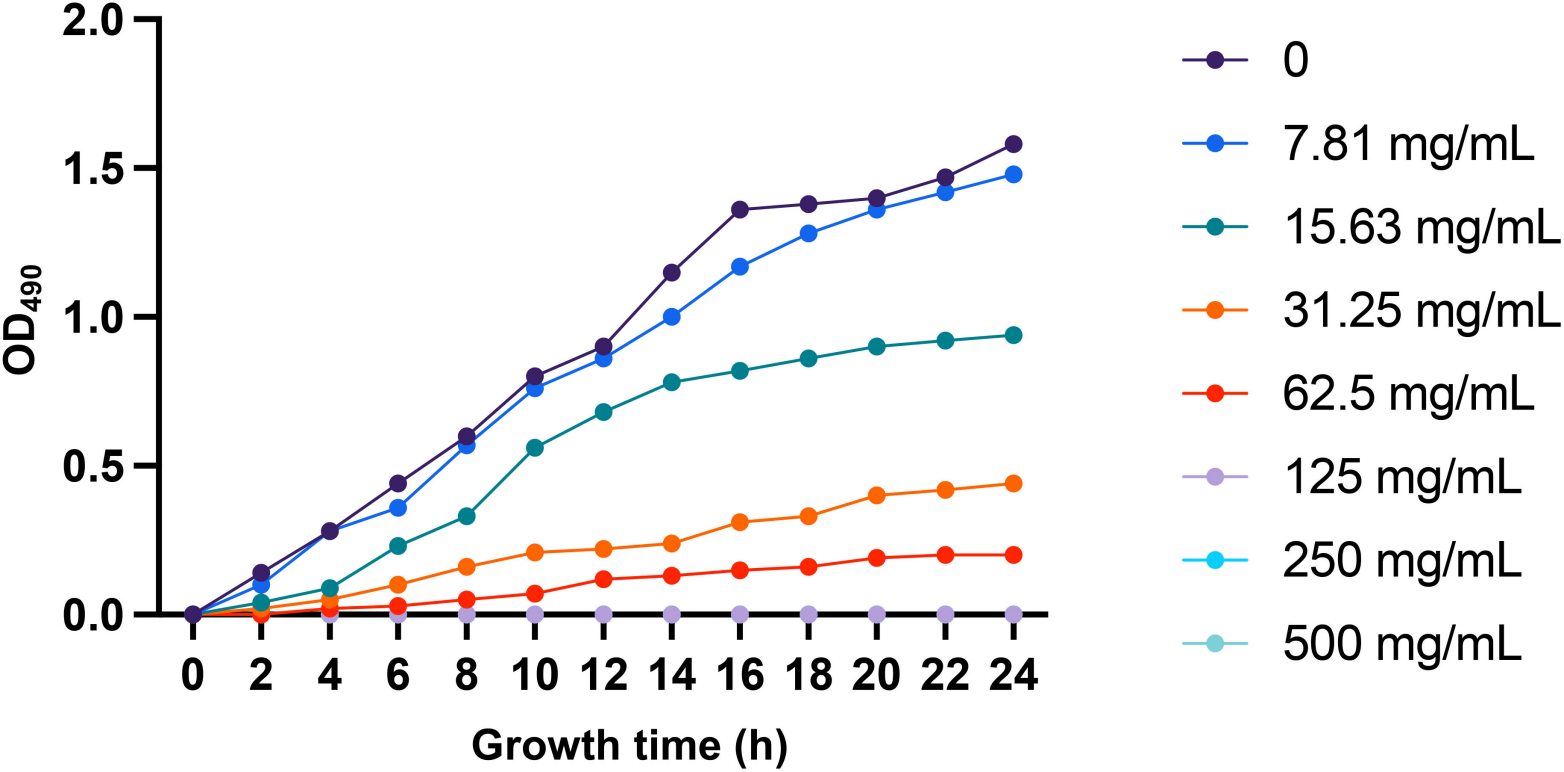
CRKP growth curve after treatment with berberine hydrochloride. The CRKP strains were cultured in MH broth for 24 hours, and bacterial growth was monitored by measuring the optical density (OD) at 490 nm at intervals of 2 hours. The growth of the plants in the treatment groups exposed to varying concentrations of berberine hydrochloride was inhibited. The MIC of berberine hydrochloride against CRKP strains was determined to be 125 mg/mL, indicating effective inhibition of the growth of CRKP strains at this concentration under the tested conditions.

Previous studies have demonstrated that berberine hydrochloride exhibits antimicrobial activity against various resistant bacteria, including *Staphylococcus aureus*, *Pseudomonas aeruginosa*, and *Cutibacterium acnes* (Chu et al., 2016; Sun et al., 2023; Zhou et al., 2023; Liu et al., 2024). In our *in vitro* experiments, we determined the minimum inhibitory concentration (MIC) of berberine hydrochloride against CRKP strains to be 125 mg/mL (**Figure 2**). At concentrations of 125 mg/mL and above, berberine hydrochloride significantly inhibited CRKP growth, leading to complete bacterial elimination (**Figure 2**). After the broth was transferred to agar plates and incubated for 24 h, no bacterial growth was observed, confirming the bactericidal effect. These findings suggest that berberine hydrochloride has potent inhibitory effects on CRKP *in vitro*, supporting its potential as a therapeutic agent against these resistant strains.

## Discussion

*Klebsiella pneumoniae* (KP), the second most prevalent Gram-negative pathogen in China, poses escalating threats due to rising carbapenem resistance (CRKP), particularly in bloodstream infections and immunocompromised populations. CRKP-associated blood stream infections often correlate with high mortality, prolonged hospitalization, and therapeutic failure, necessitating sustained surveillance in high-risk settings like ICUs (Zhang et al., 2018). Unlike our previous multisource CRKP analysis (Zhu et al., 2022), this study specifically characterizes 94 bloodstream infections derived CRKP strains, revealing near-universal multidrug resistance linked to nosocomial clustering and antibiotic overuse. Notably, polymyxin retained efficacy (5.32% resistance rate), though emerging resistance underscores the urgency for judicious use. These findings mandate three clinical priorities: (1) enhanced bloodstream infections focused CRKP surveillance to track resistance evolution in ICUs and transplant units; (2) restricted polymyxin deployment guided by rapid susceptibility testing to preserve its efficacy; and (3) implementation of antibiotic stewardship programs targeting carbapenem and cephalosporin overuse to mitigate multidrug resistance amplification. Future research should prioritize combinatorial therapies leveraging polymyxin’s residual activity while exploring alternative agents against resistant subpopulations.

Our findings demonstrate that carbapenemase production [predominantly *bla*_KPC_ (62.70%) and *bla*_NDM_ (14.89%)] remains the primary resistance mechanism in CRKP bloodstream infectious, contrasting with global reports highlighting *bla*_CTX-M_ prevalance in extended-spectrum beta-lactamase (ESBL)-mediated resistance (Scheuerman et al., 2018). Notably, the convergence of antimicrobial resistance and virulence markers presents critical clinical challenges. The high *fimH* gene prevalence (67.7%) in KPC-positive strains suggests enhanced mucosal adhesion and immune evasion capabilities, while capsule serotype K1/K2 and hypermucoviscous phenotypes (15.96%) in our cohort align with established hypervirulence markers (*alls*, *uge*, *wabG* genes). Paradoxically, limited biofilm formation (6.38%) and serum sensitivity (12.77%) reflect the evolutionary trade-off between virulence and resistance mechanisms (Schroeder et al., 2017; Cepas and Soto, 2020). These findings highlight the need for routine screening of *bla*_KPC_ and *bla*_NDM_ carbapenemases alongside virulence markers (e.g., *fimH*, capsule typing) to guide combination therapies. Enhanced infection control protocols targeting hv-CRKP transmission, especially for K1/K2 serotypes, are also crucial. Additionally, developing rapid diagnostics that integrate resistance genotyping and virulence profiling is essential. Future research should focus on longitudinal surveillance of hv-CRKP evolutionary and clinical validation of virulence-inhibiting adjuvants to complement existing carbapenemase-targeted therapies.

In our study, ~84% of bloodstream infections (71.28% ICU, 12.77% hematology) originated in high-risk units, reflecting the vulnerability of critically ill patients with prolonged hospitalization, immunosuppression, and invasive interventions. While established risk factors (carbapenem use, ICU admission, invasive procedures) align with prior studies (Li et al., 2020; Yuan et al., 2020; Zhang et al., 2021), we identified novel prognostic determinants: respiratory comorbidities, intravenous catheterization, and prior third-generation cephalosporin/enzyme inhibitor use independently predicted poor outcomes (Hussein et al., 2013; Brennan et al., 2014), whereas traditional Chinese medicine adjunctive therapy significantly improved prognosis. Our findings suggest the need for adjunctive therapy trials to evaluate the immunomodulatory or anti-biofilm effects of traditional Chinese medicine’s in CRKP management.

Recent studies have focused on the antibacterial properties of traditional Chinese medicine owing to its low propensity for drug resistance and broad-spectrum antibacterial effects across multiple target sites (Wojtyczka et al., 2014; Li et al., 2021). Meanwhile, treatment with traditional Chinese medicine, either alone or in combination with Western medicine, is emerging as a novel approach to combat drug-resistant bacteria. Previous studies have highlighted the synergistic effects of combining berberine hydrochloride (commonly known as berberine) with antibiotics (Wojtyczka et al., 2014; Zhou et al., 2023). According to our study results, berberine hydrochloride exhibited significant antibacterial activity *in vitro*, with an MIC of 125 mg/mL against CRKP isolates, suggesting its potential for clinical use in anti-CRKP treatment. However, in-depth research in this direction is warranted to explore and develop additional Chinese medicine treatment options for patients with multidrug-resistant CRKP infections. Overall, our study highlights the potential of traditional Chinese medicine as a valuable resource for addressing the challenges posed by drug-resistant bacteria.

### Limitations of the study

Despite several significant findings, a few limitations of the present study should be acknowledged. First, the relatively small sample size may have affected the analysis of risk factors. Second, the underlying mechanism by which berberine hydrochloride affects CRKP strains, particularly multidrug-resistant strains, still needs to be investigated. Therefore, future research with larger sample sizes and focused mechanistic exploration is warranted to address these limitations, thereby providing a more comprehensive understanding of CRKP infections and their treatment options.

## Data Availability

The original contributions presented in the study are included in the article/**Supplementary Material**. Further inquiries can be directed to the corresponding author.
