# Supplementary material for: Characterization of carbapenem-resistant Klebsiella pneumoniae in bloodstream infections: antibiotic resistance, virulence, and treatment strategies
Source: Front Cell Infect Microbiol. 2025 Mar 7;15:1541704. doi: 10.3389/fcimb.2025.1541704 (PMC11925884; doi:10.3389/fcimb.2025.1541704)
Supplement: Supplementary file 1 [file DataSheet1.pdf]

Supplementary Materials for

Characterization of Carbapenem-Resistant *Klebsiella pneumoniae* in Bloodstream Infections: Antibiotic Resistance, Virulence, and Treatment Strategies

Chenglin Zhong[1#], Shaohua Lin[1#], Zeqi Li[1#], Xuejing Yang[1*]

Affiliations:

1 The First Affiliated Hospital of Zhejiang Chinese Medical University (Zhejiang Provincial Hospital of Traditional Chinese Medicine), Hangzhou, Zhejiang 310006, P.R. China.

# Contributed equally.

*Corresponding author: Xuejing Yang. E-mail: yxjyxj2006@126.com， Department of Clinical Laboratory，the First Affiliated Hospital of Zhejiang Chinese Medical University(Zhejiang Provincial Hospital of Traditional Chinese Medicine)，54 Youdian Road, Hangzhou, Zhejiang 310006, P.R. China.

Table S1 Primers for carbapenem-resistant genes detection.

Gene names	Primer sequences (5’-3’)	Length (bp)
KPC	F: CGTCTAGTTCTGCTGTCTTG	798
	R: CTTGTCATCCTTGTTAGGCG	
NDM	F: GGTTTGGCGATCTGGTTTTC	621
	R: CGGGAATGGCTCATCACGATC	
VEB	F: ATGCAAAGCGTTATGAAATTTCCG	553
	R: CTGCTGCAATTCCATTTATTTATCC	
OXA-48	F: GCGTGGTTAAGGATGAACAC	438
	R: CATCAAGTTCAACCCAACCG	
GES	F: ATGCGCTTCATTCACGCAC	864
	R: CTATTTGTCCGTGCTCAGG	
IMP	F: GGAATAGAGTGGCTTAAYTCTC	232
	R: GGTTTAAYAAAACAACCACC	
VIM	F: ATGTTAAAAGTTATTAGTAGT	801
	R: CTACTCGGCGACTGAGCGAT	

Note: F represents the forward primer, and R represents the reverse primer.

Primers mentioned above for carbapenem-resistant genes detection were designed from the reference (1).

Table S2 Detailed PCR reaction conditions for carbapenem-resistant genes detection.

Reaction stage	Reaction temperature	Reaction time	Cycle numbers
Initial degeneration	95℃	6 min	1
Denaturation	95℃	30 s	
Annealing	X	30 s	30
Extension	72℃	Y	
Final extension	72℃	7 min	1

Note: PCR conditions include initial denaturation, denaturation, annealing, and extension steps with specified temperatures and durations. The annealing temperature X and extension time Y were adjusted according to the sequence and fragment length of the primers for each gene.

Table S3 Primers for virulence-associated genes detection.

Name of genes	Primer sequence (5'-3')	Length (bp)
K1	F: GGTGCTCTTTACATCATTGC<br>R: GCAATGGCCATTTGCGTTAG	1283
K2	F: GACCCGATATTCATACTTGACAGAG<br>R: CCTGAAGTAAAATCGTAAATAGATGGC	641
K5	F: TGGTAGTGATGCTCGCGA<br>R: CCTGAACCCACCCCAATC	280
K20	F: CGGTGCTACAGTGCATCATT<br>R: GTTATACGATGCTCAGTCGC	741
K54	F: CATTAGCTCAGTGGTTGGCT<br>R: GCTTGACAAACACCATACAG	881
K57	F: CTCAGGGCTAGAAGTGTCAT<br>R: CACTAACCCAGAAAGTCGAG	1037
fimH	F: TGCTGCTGGGCTGGTCGATG<br>R: CGGAGGGTGACGGTGACATC	909
mrkD	F: TTCTGCACAGCGGTCCC<br>R: GATACCCGGCGTTTTCGTTAC	246
magA	F: GGTGCTCTTTACATCATTGC<br>R: GCAATGGCCATTTGCGTTAG	1100
rmpA	F: ACTGGGCTACCTCTGCTTCA<br>R: CTTGCATGAGCCATCTTTCA	516
alls	F: CCGAAACATTACGCACCTTT<br>R: ATCACGAAGAGCCAGGTCAC	508
wabG	F: ACCATCGGCCATTTGATAGA<br>R: CGGACTGGCAGATCCATATC	683
uge	F: TCTTCACGCCTTCCTTCACT<br>R: GATCATCCGGTCTCCCTGTA	534
aerobactin	F: GCATAGGCGGATACGAACAT	556

	R: CACAGGGCAATTGCTTACCT	
iroN	F: GTCCGGCGGTAACTTCAGCC	829
	R: TCAGAATGAAACTACCGCCC	
iutA	F: GGCTGGACATCATGGGAACTGG	300
	R: CGTCGGGAACGGGTAGAATCG	
entB	F: ATTTCCTCAACTTCTGGGGC	371
	R: AGCATCGGTGGCGGTGGTCA	

Note: F represents the forward primer, and R represents the reverse primer.

Primers mentioned above for virulence-associated genes detection were designed from the reference (2).

Table S4 Detailed PCR reaction conditions for virulence-associated genes.

Name of genes	Annealing temperature (℃)	Extension time (s)
K1	55	70
K2	55	45
K5	55	35
K20	55	45
K54	55	55
K57	53	55
aerobactin	60	40
rmpA	60	40
magA	55	90
iutA	60	30
uge	55	40
wabG	56	30
alls	58	45
iorN	60	50
wcaG	53	30
fimH	55	55
mrkD	50	30
entB	52	30

Note: Crucial PCR conditions include the annealing temperature, and extension time for each virulence-associated gene were listed as above.

Supplementary references

1. R. R. Makharita, I. El-Kholy, H. F. Hetta, M. H. Abdelaziz, F. I. Hagagy, A. A. Ahmed and A. M. Algammal: Antibiogram and Genetic Characterization of Carbapenem-Resistant Gram-Negative Pathogens Incriminated in Healthcare-Associated Infections. *Infect Drug Resist*, 13, 3991-4002 (2020) doi:10.2147/idr.S276975
2. E. D. Candan and N. Aksöz: Klebsiella pneumoniae: characteristics of carbapenem resistance and virulence factors. *Acta Biochim Pol*, 62(4), 867-74 (2015) doi:10.18388/abp.2015_1148
